# RE: Could Hepatitis B Reactivation Be Overlooked in Patients with Resolved HBV Infection and Receiving Immunosuppressive Treatment?

**DOI:** 10.5152/tjg.2024.235342

**Published:** 2024-04-01

**Authors:** Pingyang Han, Ziqiu Wang, Zhaohui Wang

**Affiliations:** 1 Department of Nephrology, Ruijin Hospital, Shanghai Jiao Tong University Faculty of Medicine, Shanghai, China; 2 Department of Nephrology, The Affiliated Hospital of Hangzhou Normal University, Hangzhou, China; 3 Shanghai Jiao Tong University Faculty of Medicine, Shanghai, China

Dear Editor,

We are very glad to have Acar and Eminler^1^ who put forward valuable comments and suggestions for our research titled “Risk of Hepatitis B Reactivation in Patients with Resolved Infection on Therapy with Corticosteroids and Conventional Synthesis Immunosuppressants for Kidney Disease: A Single-Center Analysis of 258 Patients”.^2^ We have read this letter^1^ carefully and thank the authors for their questions and views.

The rate of hepatitis B virus (HBV) infection is relatively high in China. According to the Polaris International Epidemiology Cooperation Organization, HBsAg prevalence in the general population was 6.1% and, there were 86 million chronic HBV infections in 2016.^3^ In 2005, the Chinese Association of Infectious Diseases Branch and Hepatology Branch organized relevant domestic experts to formulate the “Guidelines for the Prevention and Treatment of Chronic Hepatitis B”, and updated it in 2010, 2015, 2019 and 2022. The latest Chinese guideline^4^ recommended the prophylactic antiviral therapy to patients with a previously resolved HBV (prHBV) infection (HBsAg-negative/anti-HBc-positive, undetectable serum HBV DNA) only if the patient was receiving B lymphocyte monoclonal antibodies, undergoing hematopoietic stem cell transplantation, or with advanced liver fibrosis/cirrhosis. The guidelines of American Association for the Study of Liver Disease (AASLD) 2015^5^ and Asian-Pacific Association for the study of the liver (APASL) 2021^6^ were consistent with the Chinese guideline. The patients with a prHBV infection who received cytotoxic chemotherapy (except anthracyclines) and steroid (high dose) ≥20 mg/day have the low risks of HBVr (<1%). For those with low risk of HBVr, initiation of pre-emptive nucleos(t)ide analogues (NUCs) depends on whether the patients had advanced fibrosis or cirrhosis. If the patients had advanced fibrosis or cirrhosis, pre-emptive NUCs should be initiated. Comparatively, those with no advanced fibrosis or cirrhosis need to have a serum ALT monitored every 3 months. When the ALT were detected to elevate more than twice of the baseline, HBsAg and HBV DNA should be performed and high-resistant barrier NUCs initiated if either test positive. These findings reinforce the previous reports.

Reactivation of hepatitis B is defined by a significant increase in HBV replication usually accompanied by elevations in serum aminotransferase levels and sometimes by jaundice.^5^ Our manuscript was described a retrospective study of real cases, of which 41.1% monitored serum HBV markers and HBV DNA every 1-3 months after the initiation of immunosuppressive therapy, while the remaining patients only monitored liver function. Although there are a few defects in this treatment, no significant statistical differences between the two groups of patients in the clinical features and immunosuppressive therapies makes up for this deficiency to a considerable extent.

In this study, a total of 9 patients were considered to exhibit seroconversion of anti-HBs from negative to positive, among which 2 received hepatitis B vaccine during this period and another 7 had the antibody titers just around the critical value within the acceptable laboratory testing errors. In addition, the anti-HBS of the patients during the window period from acute hepatitis B infection to recovery could not be detected until several weeks to months later_._^7^

As a country with a large population, China has a large number of patients in renal and rheumatology departments to be treated with steroids or immunosuppressants. Our findings preliminarily suggested that the prHBV infection patients receiving steroid and immunosuppressive therapy need regular liver function, HBsAg, and HBV DNA tests, in accordance with the guidelines of 2015 AASLD, 2021 APASL, and 2022 Chinese, but not necessary to initialize NUCs therapy at present. Due to the limitations of this study, HBVr is still needed to be confirmed by prospective, large sample sizes and more uniform studies.
