# Erratum

**DOI:** 10.5152/tjg.2024.030624

**Published:** 2024-06-01

In the Author’s Response authored by Öter and Yalçın, titled “RE: What Is the Optimal Treatment for Liver Hydatid Cysts in Emergency and Elective Situations?” (Turk J Gastroenterol. 2024;35(2):160. DOI: 10.5152/tjg.2024.235342) published in the February 2024 issue of Turkish Journal of Gastroenterology, an inadvertent duplication of the DOI referencing a different Author’s Response article titled “RE: Could Hepatitis B reactivation be overlooked in patients with resolved HBV infection and receiving immunosuppressive treatment?” (Turk J Gastroenterol. 2024;35(4):352-353. DOI: 10.5152/tjg.2024.235342) occurred. Subsequently, a distinct DOI has been assigned (DOI: 10.5152/tjg.2024.235232), and the online version of the Author’s Response has been updated accordingly. You can access the revised version of this article via the following link:

https://www.turkjgastroenterol.org/en/re-what-is-the-optimal-treatment-for-liver-hydatid-cysts-in-emergency-and-elective-situations-137183

